# Association Between Severity of COVID-19 and Social Determinants of Health with Adverse Pregnancy Outcomes in a Study of Mother–Infant Pairs in Los Angeles, California

**DOI:** 10.3390/women5020012

**Published:** 2025-04-02

**Authors:** Sarah Daouk, Tara Kerin, Trevon Fuller, Olivia Man, Mary C. Cambou, Viviana Fajardo-Martinez, Sophia Paiola, Thalia Mok, Rashmi Rao, Karin Nielsen-Saines

**Affiliations:** 1 Department of Pediatrics, David Geffen School of Medicine at the University of California, Los Angeles, CA 90095, USA; 2 Institute of the Environment and Sustainability at the University of California, Los Angeles, CA 90095, USA; 3 David Geffen School of Medicine at the University of California, Los Angeles, CA 90095, USA; 4 Department of Medicine, David Geffen School of Medicine at the University of California, Los Angeles, CA 90095, USA; 5 Department of Obstetrics and Gynecology, David Geffen School of Medicine at the University of California, Los Angeles, CA 90095, USA

**Keywords:** pregnancy, adverse pregnancy outcomes, COVID-19 severity, social determinants of health, COMP cohort

## Abstract

Previous cross-sectional studies have investigated social determinants of health (SDOH) among pregnant women with COVID-19. However, there are scant data on the impact of these determinants on maternal outcomes from cohorts of pregnant women with COVID-19. We evaluated the association between social determinants of health and both COVID-19 severity and adverse pregnancy outcomes (APOs) in a cohort of pregnant women in Los Angeles (L.A.) County, California. The APOs considered were fetal loss, gestational hypertensive disorders, prolonged rupture of membranes, and maternal death. We recruited pregnant women with confirmed SARS-CoV-2 and collected data on maternal COVID-19 severity, trimester at diagnosis, comorbidities, mode of delivery, COVID-19 vaccination, APOs, maternal age, medical insurance type, race/ethnicity, and neighborhood income. Participants who were obese were more likely to experience severe COVID-19 (OR: 3.61, 95% CI: 1.44–9.46), while even one vaccine dose before COVID-19 infection was associated with reduced odds of severe disease (OR:0.14, 95% CI: 0.02–0.52). Pregnant participants living in low-income areas were more likely to experience APOs (*p* = 0.01) and severe COVID-19 (*p* = 0.009). This suggests that economic inequities could negatively impact maternal outcomes among pregnant women with COVID-19. We also found that SDOH moderated severity effects on APOs in Black women vs. non-Black women. These findings underscore the importance of considering social determinants of health to improve maternal health.

## 1. Introduction

The impact of SARS-CoV2 infection is often severe in high-risk individuals including pregnant women. Recent studies have shown that COVID-19 severity in pregnant women is linked with increase adverse pregnancy outcomes (APOs) including neonatal and maternal complications with an associated risk of maternal mortality from severe COVID-19 disease [1-6]. Pregnancy renders women more susceptible to infections, while prenatal physiological changes further contribute to adverse outcomes [7]. Understanding the factors that add additional impacts to COVID-19 severity and APOs is crucial to determining quality-informed healthcare for pregnant women.

The World Health Organization defines social determinants of health (SDOH) as “the conditions of where a person is born, where they grow up, where they live, where they work, and where they age [8]”. SODHs are ascribed to five domains: economic stability, education access and quality, healthcare access and quality, neighborhood and built environment, and social and community context [9]. In the United States, SDOH have been shown to have great influence in the acquisition of SARS-CoV-2 [10], from the impacts of economic stability (such as living in close quarters or homelessness [11]), healthcare access and quality (including insurance [12]), and social/community context (including race/ethnicity [13]). SDOH also have a known impact on pregnancy outcomes [14], with the United States having infant mortality rates that surpass other similarly developed countries [15], with drastic differences in adverse pregnant outcomes (APOs) by race/ethnicity [16], by access to healthcare [17], and by low-income neighborhoods [18]. Previous cross-sectional studies have investigated SDOH among pregnant women with COVID-19. International cohort studies evaluating the impact of SDOH on COVID-19 severity among pregnant women have suggested that the frequency of severe COVID-19 is higher among participants with lower socioeconomic status, while cross-sectional studies in pregnancy have also suggested that low income is associated with severe disease [19]. In the United States, higher maternal and infant mortality have been linked to severe COVID-19 in minorities [20], and SDOH have been linked to increases in APOs [21]; however, there are scant data on the impact of SDOH on overall maternal and infant outcomes from cohorts of pregnant women with COVID-19 in the United States, and on if SDOH and severity interact to increase APOs.

The objective of this study was to evaluate the impacts of social determinants of health on COVID-19 severity and adverse pregnancy outcomes in a prospective cohort of pregnant women followed in a health system providing medical care across the metropolitan Los Angeles area and encompassing a network of outpatient centers and two University of California, Los Angeles (UCLA) Health hospitals. We enrolled a unique cohort of pregnant women who were positive for COVID-19 in the early stages of the global pandemic. Leveraging this cohort, we examined the impact of SDOH on maternal and infant outcomes to increase the knowledge of the intersection of COVID-19, SDOH, and maternal health in the United States. We predicted that rates of APOs would be higher in the presence of SDOH, including economic disparity, race/ethnicity, and healthcare access, acting either directly or as a mediating variable. Outcomes of this study may increase information for SDOH-directed outreach for pregnant women with COVID-19.

## 2. Materials and Methods

To ensure the quality of our findings, we used the STROBE statement guidelines [22]. These guidelines were established to ensure the complete and accurate reporting of an observational study.

### 2.1. Study Design and Participants

The results presented here are a cross-sectional analysis of data from the UCLA COVID-19 Outcomes Mother–Infant Pair (COMP) prospective cohort study. The COMP study consisted of pregnant women with confirmed COVID-19 and their infants. Pregnant women aged 18 and above with SARS-CoV-2 infection confirmed by NP RT-PCR or serology at any point during gestation were eligible for enrollment at the University of California, Los Angeles. Detailed descriptions of the cohort, including eligibility requirements, consent process, data collection, and investigators, have been published previously [2-4,23,24], but, briefly, a prospective observational cohort of mother–infant dyads diagnosed with SARS-CoV-2 infection in pregnancy in L.A. County was established. Data, including biological samples from mother and child, were collected prospectively for the cohort, with maternal comorbidities retrospectively collected from medical records. Clinical pregnancy and infant outcomes were collected prospectively.

From 15 April 2020 to 30 August 2022, we recruited into the COMP study pregnant women aged 18 and older at UCLA Health outpatient obstetric clinics across Los Angeles County, during hospital admission to UCLA, and from the labor and delivery unit at the two UCLA Health hospitals. In total, 221 women and 227 infants were enrolled, and were followed until 2024. Women who tested positive for COVID-19 at any point during pregnancy by either PCR or antigen testing were approached in the outpatients setting or at Labor and Delivery at Ronald Reagan or Santa Monica College for participation in the study. Inclusion criteria included women who were pregnant, 18 or older, and admitted in one of the two UCLA Heath hospitals, and who could understand and consent to the study. Exclusion criteria consisted of an inability or unwillingness to consent to the study. All pregnancies and their resulting infants were included in the fetal outcome data. No inclusions/exclusions were made by race/ethnicity.

### 2.2. Data Collection

Clinical, obstetrical, and laboratory results for both mothers and infants were abstracted prospectively from medical records by a multidisciplinary team of infectious disease specialists, maternal–fetal medicine specialists, and neonatologists. The clinical data collected included maternal COVID-19 disease severity, trimester at diagnosis, multiple versus singleton gestation, maternal comorbidities (asthma, congenital heart diseases, substance use disorder, diabetic disorders (both preexisting diabetes and gestational diabetes), autoimmune disease, obesity), history, number of doses and timing of COVID-19 immunization, birth of a live infant, mode and timing of delivery (term/preterm), obstetrical complications, maternal and infant outcomes including the assigned sex of the neonate, birth weight, and APGAR scores (1 and 5 min).

In addition to collecting clinical data, we also captured the cross-sectional social determinant of health parameters via chart review. We included the following as proxies for SDOH: medical insurance type for healthcare access, ZIP code to determine economic neighborhood disparities, and self-reported race/ethnicity for social/community context. Using the mother’s address, we determined the median household income in her ZIP code [25]. ZIP code analysis was only performed for participants who were residents of L.A. County (85% of participants). We also captured the mother’s self-reported race/ethnicity (collapsed to the categories of Asian, Black, Hispanic/Latina, Other, and White) through a questionnaire given to participants at enrollment. Although we acknowledge that race is a social construct, we chose to include it in the analysis given the higher risk of severe and critical COVID-19 among women of color. We used L.A. County maps available through ArcGIS [26] to map the ZIP codes/median income against a base map of vector data of Los Angeles. Polygon shapefiles were retrieved from OpenStreetMap [27], an open database where data extracted after September 2012 are licensed through the terms of the Open Database License, “ODbL” 1.0 (https://opendatacommons.org/licenses/odbl/, accessed on 3 February 2025). Data are publicly available under the Open Database License.

Study data were collected and managed using REDCap electronic data capture tools hosted at UCLA [28,29]. REDCap (Research Electronic Data Capture) is a secure, web-based software platform designed to support data capture for research studies, providing (1) an intuitive interface for validated data capture; (2) audit trails for tracking data manipulation and export procedures; (3) automated export procedures for seamless data downloads to common statistical packages; and (4) procedures for data integration and interoperability with external sources [28,29]. Selected staff were given training and clearance, according to UCLA’s REDCap access requirements, to access medical record for data extraction. Data were exported in a .csv file format for analysis using SPSS 19.0 IBM Corporation, Armonk, NY, USA).

### 2.3. Operational Definitions

NIH guidelines were used to classify COVID-19 severity for our participants [30]: (a) Asymptomatic: individuals who test positive for SARS-CoV-2 but have no symptoms consistent with COVID-19 (N = 25); (b) Mild Illness: individuals who have any of the various signs and symptoms of COVID-19 but do not have shortness of breath, dyspnea, or abnormal chest imaging (N = 145); (c) Moderate Illness: individuals who show evidence of lower respiratory disease during clinical assessment or imaging and have saturation of oxygen (SpO2) > 94% on room air (N = 22); (d) Severe Illness: individuals who have SpO2 < 94% on room air, a ratio of arterial partial pressure of oxygen to fraction of inspired oxygen (PaO2/FiO2) < 300 mmHg, respiratory frequency > 30 breaths per minute, or lung infiltrates > 50% (N = 15); and (e) Critical Illness: individual who have respiratory failure, septic shock, and/or multiple organ dysfunction (N = 14).

Pregnancy was categorized into three trimesters: first trimester (0–13 weeks), second trimester (14–27 weeks), and third trimester (>28 weeks).

Covariates: Women of color were defined as women who self-reported any form of “non-white” ethnicity. However, in the analysis, race/ethnicity was evaluated categorically and included Asian, Black/African American, Hispanic/Latinx, White, and Other/Unknown.

The WHO has stated that “social determinants of health are the circumstances in which people are born, grow up, live, work and age, and the systems put in place to deal with illness” [8]. Social determinants of health can be the conditions in which people are born and grow up in and may include race, access to education, and socialization. SDOH relating to where people live may include things such as income and economic stability, access to resources such as healthcare and disease prevention, working environment safety, and the built environment. SDOH in aging may include all the above with emphasis on access to healthcare resources [8,31-34].

The primary study endpoint was an adverse pregnancy outcome (APO), defined as fetal loss, gestational hypertensive disorders (hypertension [HTN], preeclampsia, HELLP syndrome [Hemolysis, Elevated Liver Enzymes, and Low Platelets], eclampsia), prolonged rupture of membranes, or maternal death.

### 2.4. Statistical Analysis

Chi-square and Student’s *t*-tests were used when examining the association between demographics and COVID-19 severity. We performed a logistic regression in which the outcome was an APO, and the predictors were the clinical and social determinant of health variables: maternal age at enrollment, ZIP code, medical insurance type, median income, and race/ethnicity. The COVID-19 clinical categories were collapsed into asymptomatic/mild, moderate, and severe/critical for the analyses. Maternal age and household income were classified as low (below the median) and high (above the median). First, we performed univariate logistic regression for each predictor variable, then ran a full model adjusting for potential confounding variables. Confounding variables were selected based on the previous literature or because introduction of the variable to the model changed the beta by more than 10%, and included race/ethnicity, COVID-19 immunization, insurance, comorbidities (congenital heart disease, diabetes, obesity), and disease severity. The reference categories for the regression were asymptomatic/mild COVID-19, high income, high maternal age, and White ethnicity. Next, we hypothesized that SDOH moderated the effect of COVID-19 severity on APOs. To assess this, we fitted a logistic regression model in which the dependent variable was an APO and the predictors were SDOH, severity, and their interaction. The interaction term represented the extent to which SDOH moderated the severity–APO relationship, if at all. The model was fitted to the individual-level data on pregnancies resulting in live births. The SDOH variable was self-reported race, categorized as “black” or “non-black”. A two-sided hypothesis with 80% power and 5% alpha was used to determine if data were statistically significant. The probability plot was created using GraphPad Prism 10.

## 3. Results

### 3.1. Demographics

**Figure 1.**
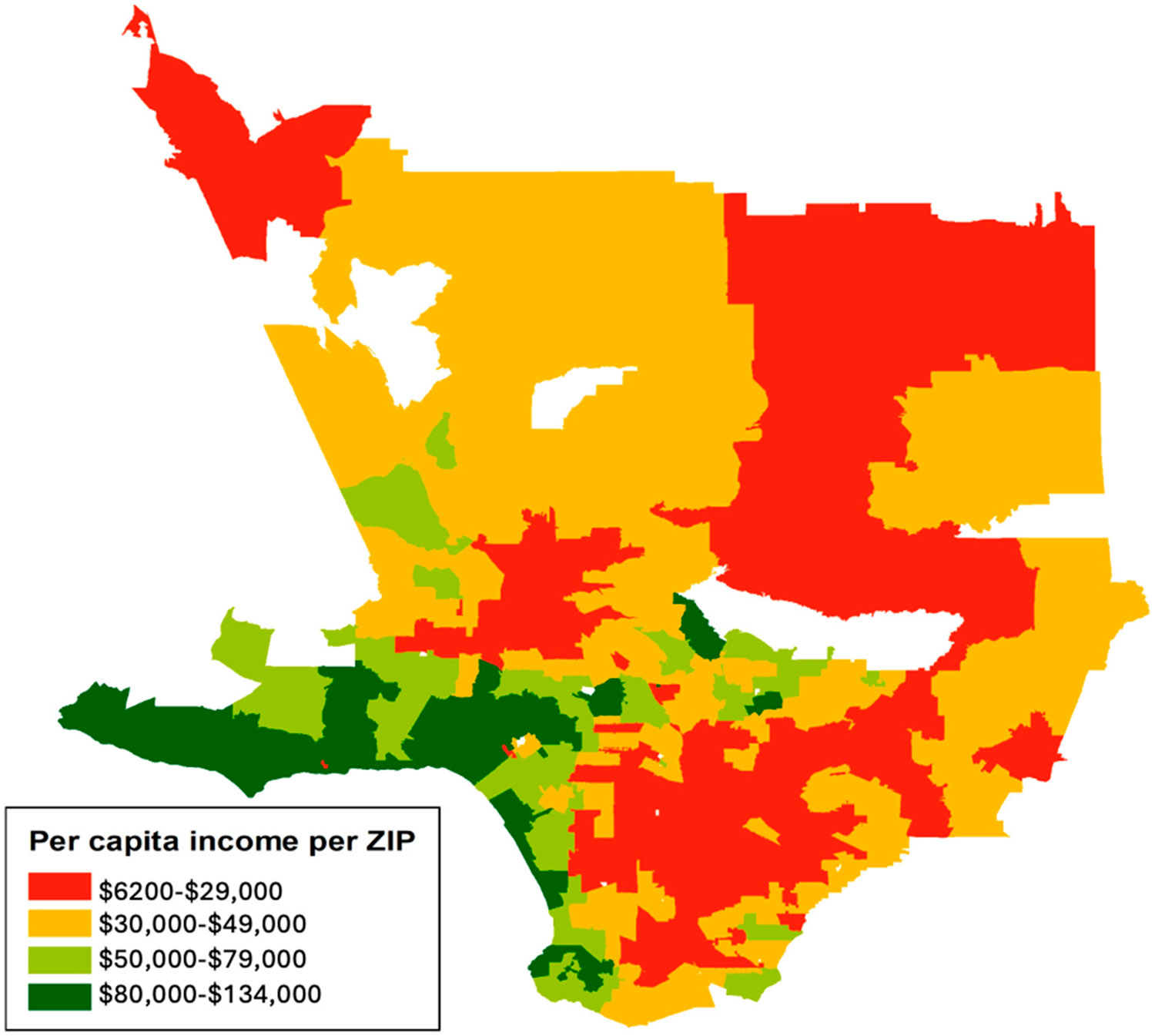
Los Angeles County map of mean household income per capita.

In total, 85% of participants resided in Los Angeles (L.A.) County and the rest lived elsewhere in Southern California. Figure 1 depicts the mean household income for the participants living in L.A. County. Table 1 describes the maternal demographic, clinical, and social characteristics of all participants. The median (Interquartile Range, IQR) maternal age in years was 33 (29–36). The most commonly self-reported race/ethnicity was Hispanic/Latina (n = 96, 43.4%), and race/ethnicity results were consistent with Los Angeles County demographics. With respect to comorbidities associated with COVID-19, 30 (13.6%) participants had asthma, 18 (8.1%) autoimmune disorders, 9 (4.1%) congenital heart disease, and 8 (3.6%) a history of substance use disorders (Table 1).

Although none of the participants received COVID-19 vaccines before pregnancy, 34.4% received at least one dose of a COVID-19 vaccine prior to delivery. Participants who received at least one dose of a COVID-19 vaccine had lower frequency of development of severe COVID-19 compared to those without vaccination (*p* = 0.01).

Parameters associated with COVID-19 severity included a lack of at least one dose of COVID-19 in pregnancy, government insurance, and obesity (Table 1). In total, 41.2% of 221 participants had at least one adverse pregnancy outcome and met a study endpoint (Table 1). Patients with hypertensive disorders of pregnancy tended to have more severe COVID-19, a finding which trended towards significance (Table 1). There was one case of maternal death followed by an intrauterine fetal demise.

### 3.2. Association of APOs and COVID-19 Severity: Influence of SODHs

When evaluating for demographic and clinical parameters potentially associated with adverse pregnancy outcomes, we did not find race/ethnicity, COVID-19 immunization, type of health insurance, maternal comorbidities, or even disease severity to be predictive of APOs in our crude and adjusted multi-variate analysis (Table 2).

An analysis of the predictors of severe COVID-19 demonstrated that at least one dose of COVID-19 vaccine was protective against severe disease in pregnancy (OR: 0.14, 95% CI: 0.02–0.53, *p* = 0.01). Obesity was again a predictor of severe disease, with OR: 3.61, 95% CI: 1.44–9.46, *p* = 0.01 (Table 3).

**Figure 2.**
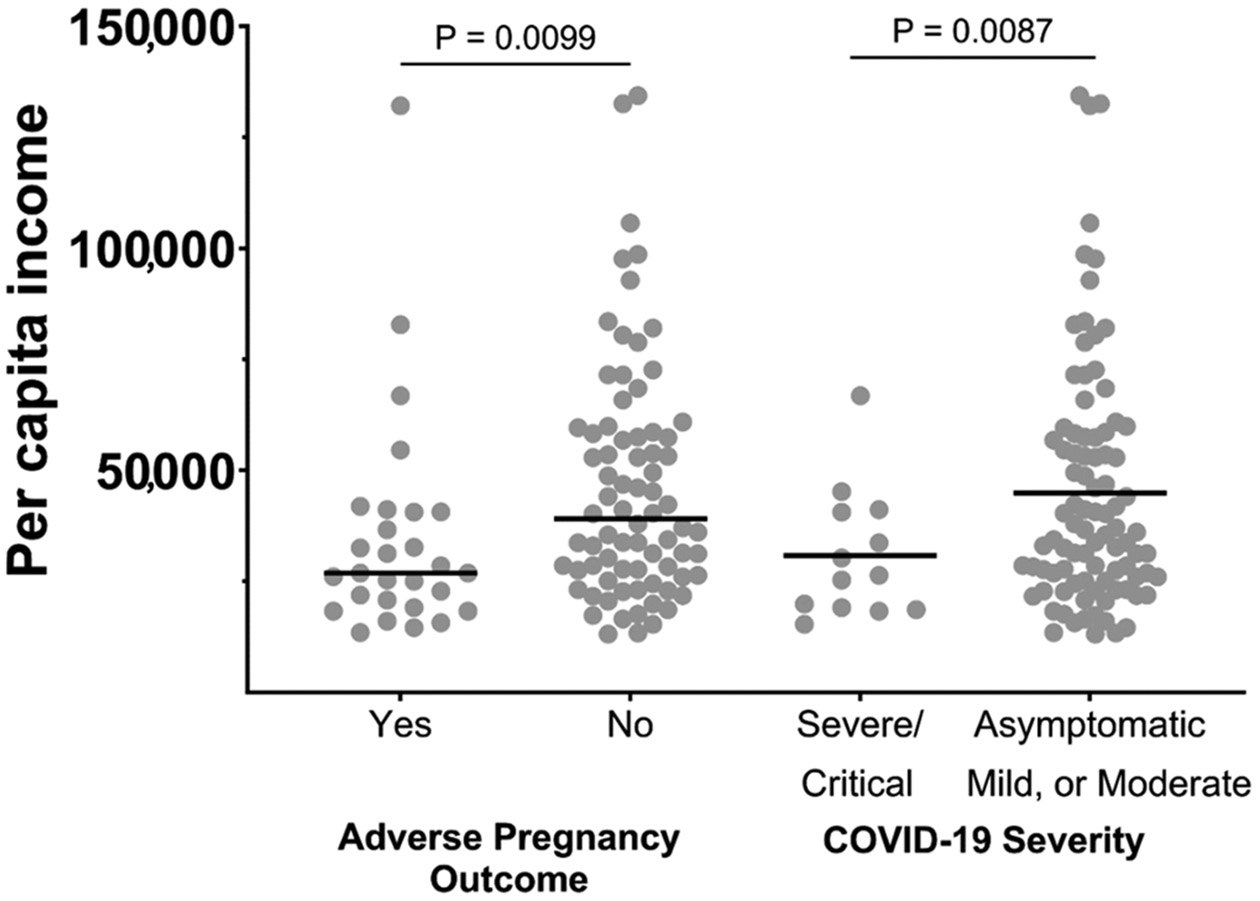
Association between per capita income, maternal COVID-19 severity, and adverse pregnancy outcomes at the ZIP code scale. (Average income was lower in ZIP codes where pregnant participants who experienced adverse outcomes resided. Income was also lower in the home ZIP codes of pregnant participants with severe or critical COVID-19. Each gray dot depicts the home zip code of pregnant participants. Horizontal bars indicate the average income per group).

**Figure 3.**
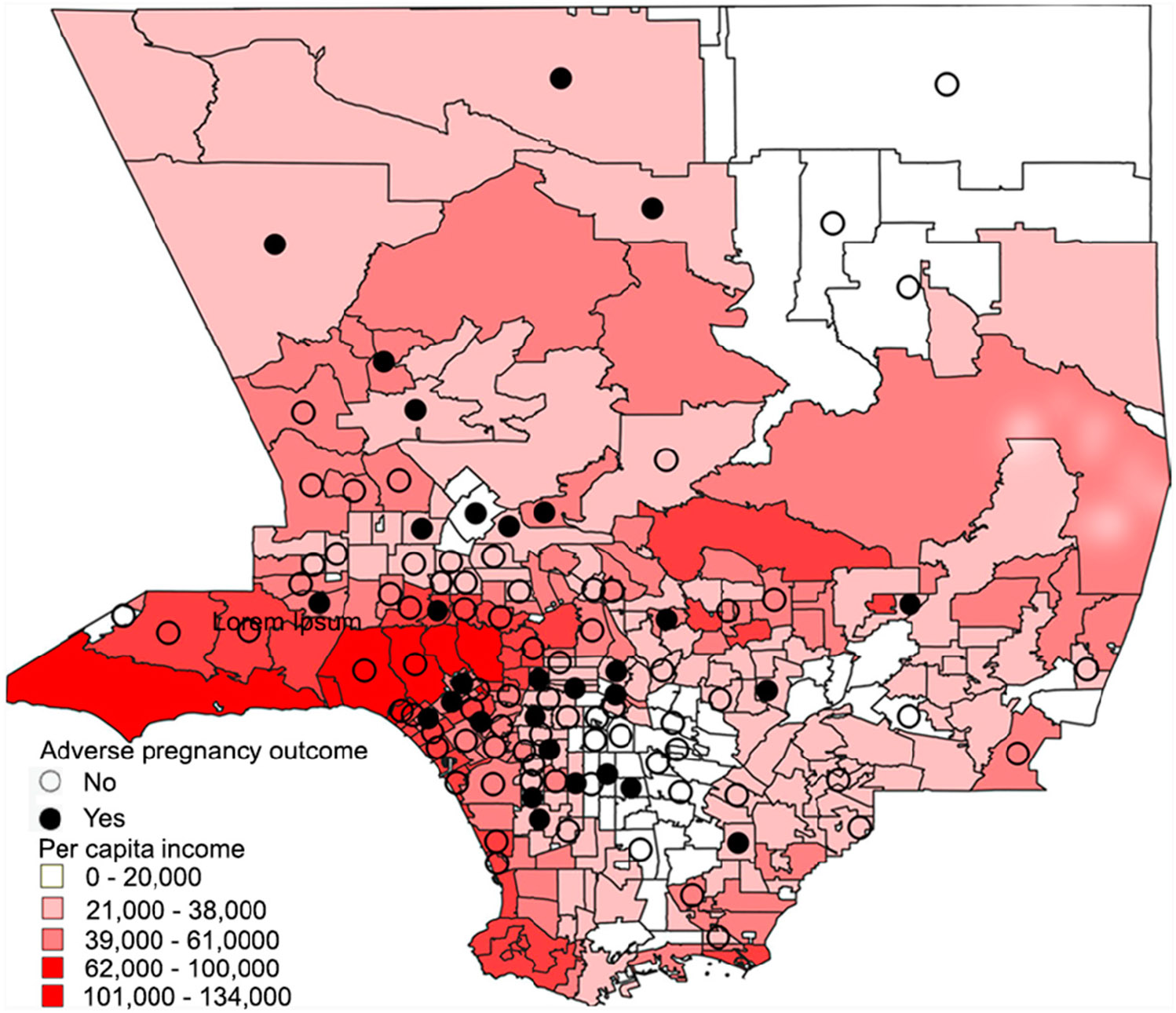
Adverse pregnancy outcomes and household income in the Los Angeles area by ZIP code. Each dot represents one pregnant participant.

**Figure 4.**
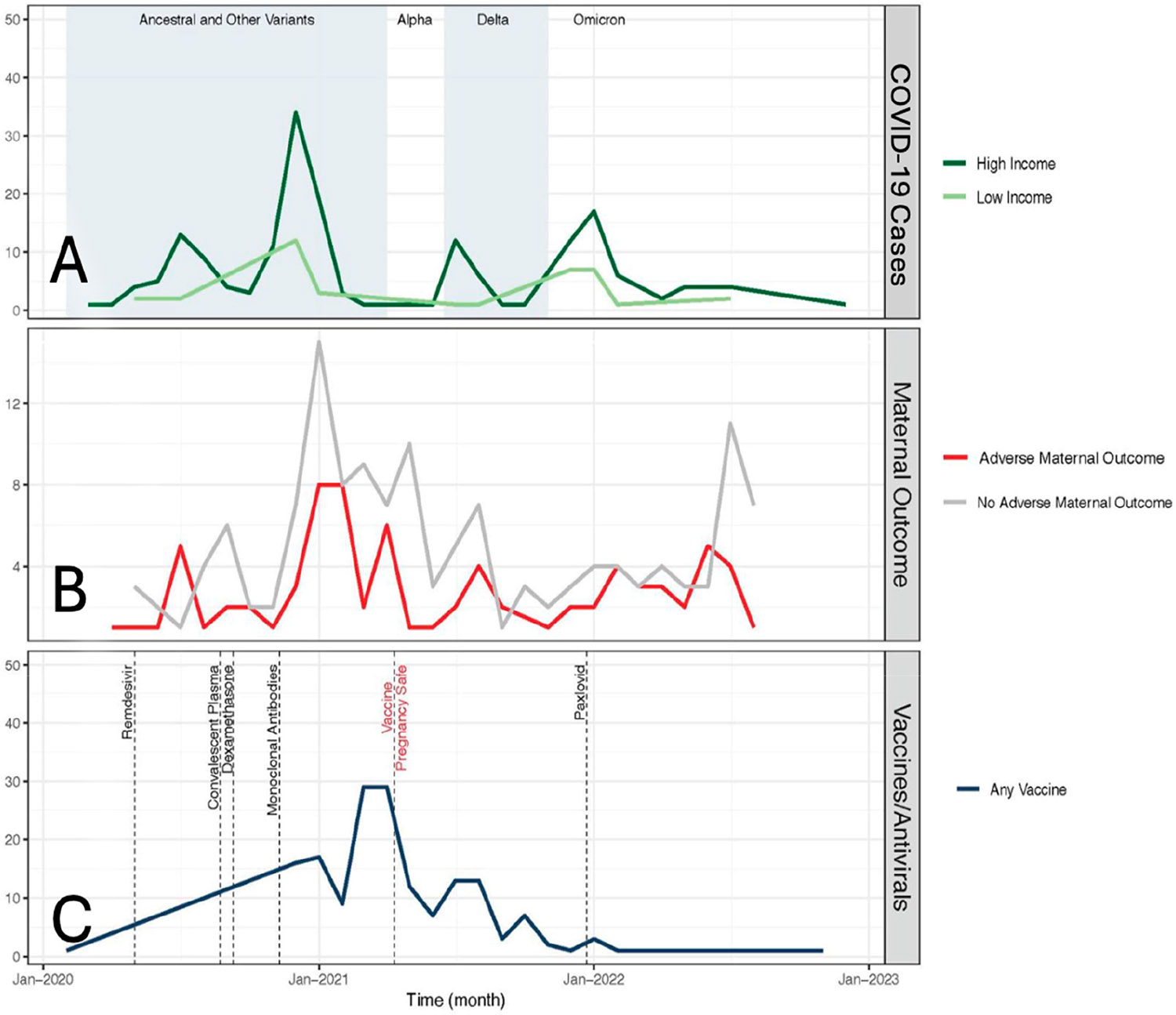
Trends over time: (**A**) COVID-19 cases among patients with high vs. low income in Los Angeles County. Alternating color/white areas represent the time when the coordinating strain was most prevalent. (**B**) Adverse pregnancy Outcomes (APO) overtime, and (**C**) Implementation of vaccines and COVID-19 treatment options available to the cohort.

**Figure 5.**
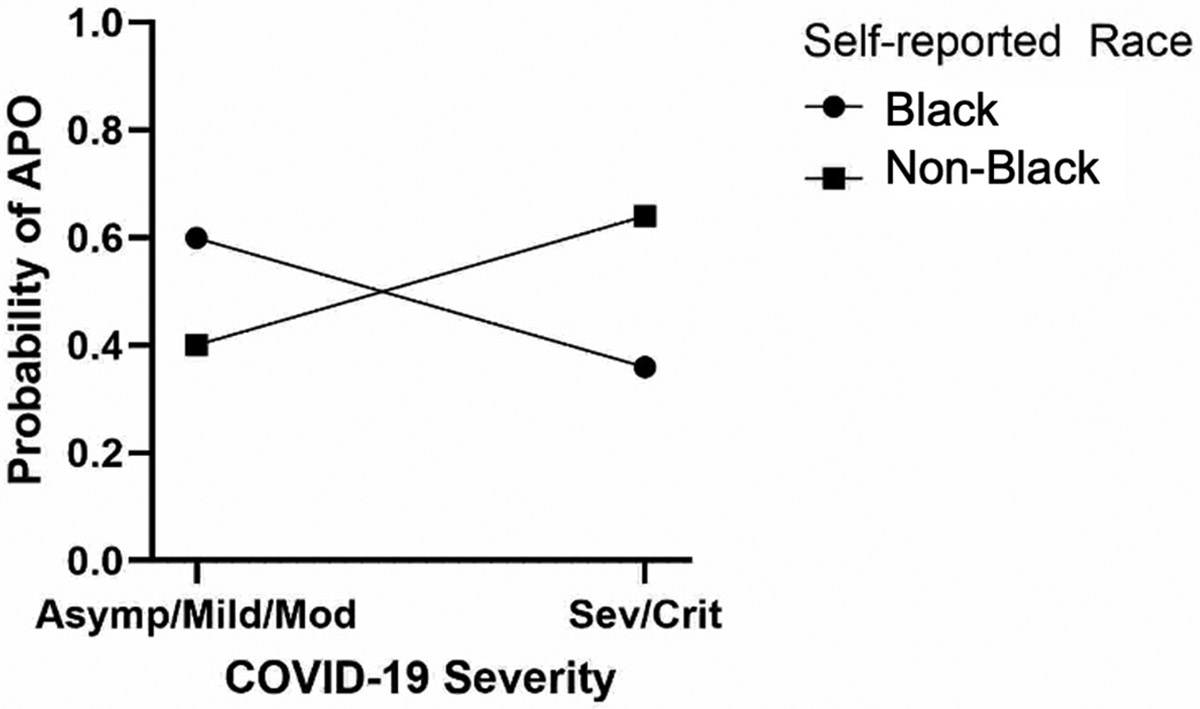
SDOG moderates the effect of COVID-19 severity on APO.

At the ZIP code scale, participants who experienced adverse pregnancy outcomes lived in ZIP codes with a lower average income, as seen in Figure 2. The mean average income was lower in ZIP codes where pregnant participants with adverse outcomes resided as compared to those with no APOs (USD 34,790 vs. USD 45,897, *p* = 0.0099). The mean income was also lower in the ZIP codes of pregnant participants with severe/critical COVID-19 versus those with milder disease: USD 35,698 vs. USD 43,989, *p* = 0.0087 (Figure 2). Adverse pregnancy outcomes tended to cluster geographically in lower-income areas in Los Angeles County, as seen in Figure 3. In total, by ZIP code analysis, 77 (35%) pregnant patients were in the higher-income category (income of USD 33,775 or higher) and 115 (52%) were in the lower-income category (13% lived outside Los Angeles County). Figure 4 demonstrates the temporal trends of COVID-19 cases in pregnant patients over time according to variants of concern and income status (4a), according to the presence or absence of adverse pregnancy outcomes (4b), and according to therapeutic developments over time (4c). Figure 5 shows the moderation effects of SDOH with COVID-19 on adverse pregnancy outcomes. African American women with a mild case of COVID-19 were more likely to experience an APO than White women with similar severity of disease. The moderation analysis identified a significant effect of severity on APOs (*p* = 0.008). The interaction term was also significant (*p* = 0.032, Figure 5). A stratified analysis by COVID-19 severity found that participants who experienced non-severe COVID-19 who self-identified as Black/African American women were 4.14 times as likely to experience an APO than White women (95% CI: 1.02, 21.22) (Table 4).

## 4. Discussion

In this study, we examined the role of social determinants of health in the development of adverse pregnancy outcomes. Surprisingly, our findings did not show a significant association between APOs and insurance. However, we saw an interaction effect with race and COVID-19 severity, where Black women were more than 4 times as likely to have an APO compared to White women with the same COVID-19 disease severity. Additionally, in our ZIP code analysis, we saw that, in areas with low median household income, there was a significant association between income and APOs, and those areas also had a significantly higher number of cases of severe COVID-19. Often, areas with low household income have been linked to overcrowding. Per the California Department of Housing and Development, the rate of overcrowding in households with very low household incomes is high [35]. Overcrowding leads to an increased rate of infectious disease spread, contributes to stress, and also carries an increased risk of exposure to secondhand smoke, all of which can adversely impact health outcomes [36]. Lower income levels have also been associated with fewer prenatal visits, which can contribute to APOs [21]. Interestingly, we did not see an association between adverse pregnancy outcomes and disease severity in our logistic regression analysis, nor was there an association between APOs and demographics, insurance status, vaccination parameters, or even maternal comorbidities. This may be due to the very high frequency of APOs in our study population, with 41% of patients having at least one APO. Other studies reported an increased risk of adverse pregnancy outcomes in patients with COVID-19 compared to pregnant individuals who did not have COVID-19 [37]. We postulate that, given the high frequency of APOs in pregnant individuals in our study, we were not able to observe significant difference in outcomes in patients with mild and severe disease.

Our study assessed the direct effect of COVID-19 severity on adverse pregnancy outcomes, as well as the effect moderation by SDOH. In this population, pregnant individuals who self-identified as Black had a higher risk of adverse pregnancy outcomes than other participants when experiencing asymptomatic, mild, or moderate COVID-19 infection. While the observational study design does not permit attributing robust casual relationships, these findings are suggestive of the moderating role of SDOH in clinical outcomes among individuals with COVID-19 infection during pregnancy.

The COVID-19 pandemic significantly exacerbated long-standing social, economic, and health inequities, with L.A. County being responsible for 1/3 of all reported cases and 36% of all COVID-19-related deaths in the state of CA, with non-Whites being more likely to contract SARS-CoV-2 [38-40]. Health inequities in maternal morbidity increased among non-White pregnant patients during the pandemic [41], with an increase in adverse pregnancy outcomes noted [42]. Studies of pregnant and non-pregnant populations during the pandemic demonstrated the negative effect of social and economic disparities on individuals diagnosed with COVID-19 [10]. Los Angeles County represents a unique location for the examination of inequities in maternal–infant health as it is home to one in every four Californians and the most populous county in the U.S., with over 10,000,000 inhabitants. It is one of the most ethnically diverse counties in the U.S., with a population that is 48% Latino, 8% Black, 15% Asian, 26% White, and 3% other [43]. Studies in other areas of the U.S. similarly demonstrated that numbers of COVID-19 hospitalizations and deaths early in the pandemic were highest in the Bronx, the area that has the highest number of ethnic/racial minorities and lowest median household income compared to other areas in New York City [44]. The INTERCOVID multinational cohort study demonstrated an increased risk of adverse pregnancy outcomes in individuals with COVID-19 as compared to uninfected pregnant controls [1]. In this study, adverse pregnancy outcomes included preeclampsia, eclampsia, HELLP syndrome, an ICU stay, and preterm delivery [1]. In a multinational cohort study evaluating social determinants of health and COVID-19 severity in pregnant individuals, younger age, lower socioeconomic class, lower educational attainment, and employment status were associated with higher odds of more severe COVID-19 [19]. However, our results did not suggest an association between APOs and race/ethnicity. This may be because, while income inequalities among minorities are still significant, there are also several pockets of higher SES minority neighborhoods [45], which may skew our findings on the association between race/ethnicity and APOs, and, instead, associations between SDOH are more evident when using ZIP code as a proxy for economic status and cultural/social constructs. We noted in our study higher rates of severe COVID-19 in pregnant patients who were not vaccinated against COVID-19, with a protective effected noted even with only one dose of vaccine prior to delivery. Our findings corroborate prior studies demonstrating the protective role of COVID-19 vaccines in pregnancy [24,46].

While we feel our study was robust, there were also some limitations. We were unable to obtain education status data and income level, nor did we have any data on social supports or built environments, which would have increased our indicators of SDOH. Additionally, all participants were followed through the UCLA Health System, and many participants may have been referred by other medical areas for complications during pregnancy, which could be responsible for the high frequency of APOs noted. Because all women approached agreed to be in the study, we do not suspect selection bias. However, because these women were seeking care, our source population may have some bias, though we suspect this would be towards the null. Although the APOs were associated with higher odds of patients self-reporting as Black, this finding was not statistically significant, likely due to the smaller proportion of Black participants in our study, which is reflective of L.A. demographics and our health system catchment area. However, race did appear to significantly impact APOs when analysis was stratified by COVID-19 severity. The ecological use of ZIP code as a proxy for income could introduce bias, but we suspect that the bias would be non-differential or a bias towards the null due to gentrification [47]. Additionally, due to sample size, we collapsed several race/ethnicities, which would benefit from being updated using current and more specific USA race classification in future studies, to be able to include more specific combinations of race and ethnicity for self-reporting. We also did not see an association between type of insurance and APOs. This may indicate that insurance was not a good proxy for healthcare access in this study. Additionally, many self-employed individuals who have a higher-than-average income may present with no insurance or government insurance and pregnant individuals in California qualify for emergency government insurance. For this reason, we do not believe that the type of insurance was as tightly associated with income as the ZIP code analysis was. While our frequencies of insurance type were similar to the national averages [48], insurance type may not have been the barrier to healthcare access during the early days of the COVID-19 pandemic. Impediments to receiving COVID-19 care during the pandemic may have been the long wait times for care, restrictions of time off work, and other factors that were affected by SDOH disparities that did not include insurance type [49]. Nevertheless, we still noted that maternal outcomes in our study population were impacted by social determinants of health, and that SDOH were moderators of COVID-19 in affecting adverse pregnancy outcomes.

## 5. Conclusions

Our findings show that disparities due to SDOH impacted pregnancy outcomes in children exposed to SARS-CoV-2 in utero, and SDOH additionally moderated the effects of COVID-19 on those outcomes. Given these findings, it is crucial to advocate for patients from lower socioeconomic backgrounds and encourage health policies that help mitigate factors that place patients at higher risk of pregnancy complications. Further studies should be dedicated to addressing local factors in Los Angeles County that contribute to the noted healthcare disparities, so that policies to curtail these inequities can be successfully implemented and hopefully remedy the social injustices that continue to plague the care of vulnerable pregnant patients.

## Figures and Tables

**Table 1.** Maternal demographics and clinical characteristics.

Pregnant Participants	All Participants		Asymptomatic/Mild/ Moderate COVID-19 N = 192		Severe/Critical COVID-19 N = 29		*p*-Value
	N	%	N	%	N	%	
Mean maternal age (std)	32.7	6.52	32.8	6	32.1	5.54	0.58
Maternal ethnicity							
Asian	28	12.7	26	13.5	2	6.9	
Black/African American	14	6.3	11	5.7	3	10.3	
Hispanic/Latinx	96	43.4	79	41.1	17	58.6	0.27
White	55	24.9	50	26.0	5	17.2	
Other/unknown	28	12.7	26	13.5	2	6.9	
COVID-19 immunization							
No dose before delivery	145	65.6	119	62.0	26	89.7	
At least one dose before infection	68	30.8	66	34.4	2	6.9	0.01
At least one dose before delivery	76	34.3	73	38.0	3	10.3	
Insurance							
Private insurance	133	60.2	118	61.5	15	51.7	
Government insurance	54	24.4	41	21.4	13	44.8	0.01
Uninsured/unknown	34	15.4	33	17.2	1	3.4	
Selected maternal comorbidities							
Asthma	30	13.6	23	12.0	7	24.1	0.07
Autoimmune disorders	18	8.1	16	8.3	2	6.9	0.79
Congenital heart disease	10	4.5	9	4.7	1	3.4	0.76
Diabetes	44	19.9	39	20.3	5	17.2	0.69
Obesity	62	28.1	45	23.4	12	41.4	<0.001
Substance abuse disorder	8	3.6	8	4.2	0	0.0	0.11
Adverse pregnancy outcomes (N = 207)							
Hypertensive Disorder of pregnancy (preeclampsia, HELLP *, gestational HTN and chronic HTN)	75	33.9	61	31.8	14	48.3	0.08
Prolonged rupture of membranes	15	6.8	14	7.3	1	3.4	0.44
Fetal loss or demise	6	2.7	5	2.6	1	3.4	
Maternal death	1	0.5	0	0.0	1	3.4	
Any adverse outcome	91	41.2	75	39.1	16	55.2	0.1

* HELLP: hemolyis, elevated liver enzymes, low platelet count; HTN: hypertension.

**Table 2.** Potential parameters associated with adverse pregnancy outcomes (APOs).

Variable	OR	Crude 95% CI		*p*-Value	OR	Adjusted * 95% CI		*p*-Value
Ethnicity/race								
White	REF							REF
Asian	0.71	0.26	1.83	0.49	0.70	0.26	1.85	0.48
Black/African American	2.70	0.82	9.81	0.11	2.62	0.75	10.16	0.14
Hispanic/Latinx	1.07	0.55	2.12	0.84	0.93	0.46	1.91	0.85
Unknown/other	0.97	0.38	2.45	0.95	1.06	0.39	2.83	0.91
COVID-19 immunization								
No dose before delivery	REF							REF
≥1 dose before infection	0.98	0.52	1.68	0.83	1.09	0.58	2.06	0.78
≥1 dose before delivery	0.74	0.01	1.11	0.13	0.18	0.01	1.18	0.13
Insurance								
Private insurance	REF							REF
Government insurance	1.51	0.80	2.86	0.20	1.27	0.63	2.57	0.51
Uninsured/unknown	0.72	0.32	1.57	0.42	0.73	0.30	1.69	0.47
Selected maternal comorbidities **								
Any	1.18	0.69	2.03	0.54	0.96	0.54	1.71	0.90
CHD	1.45	0.39	5.37	0.56	1.30	0.32	5.19	0.71
DM	1.38	0.70	2.72	0.35	1.41	0.68	2.92	0.36
Obesity	1.15	0.63	2.07	0.66	0.88	0.44	1.73	0.71
Disease Severity								
Asymp/mild/moderate	REF							REF
Severe	1.92	0.88	4.28	0.10	1.75	0.75	4.15	0.20

* Adjusted for race/ethnicity, COVID-19 immunization, insurance, comorbidities, and disease severity.

** CHD: congenital heart disease; DM: diabetes mellitus; REF: reference; Asymp: asymptomatic.

**Table 3.** Potential parameters associated with COVID-19 severity.

Variable	OR	Crude 95% CI		*p*-Value	OR	Adjusted * 95% CI		*p*-Value
Ethnicity								
White (Ref)	REF							REF
Asian	0.77	0.11	3.84	0.76	0.83	0.11	4.46	0.83
Black/African American	2.73	0.50	12.93	0.21	1.78	0.28	10.19	0.52
Hispanic/Latina	2.15	0.79	6.88	0.16	1.78	0.57	6.44	0.35
Unknown/other	0.77	0.11	3.84	0.76	0.51	0.07	2.74	0.45
COVID-19 immunization								
No dose before delivery	REF							REF
≥1 dose before infection	0.14	0.02	0.48	0.01	0.14	0.02	0.53	0.01
≥1 dose before delivery	0.65	0.03	3.90	0.70	1.21	0.06	9.57	0.87
Insurance								
Private insurance	REF							REF
Government insurance	2.49	1.08	5.70	0.30	1.52	0.58	3.98	0.39
Uninsured/unknown	0.24	0.01	1.24	0.17	0.16	0.01	0.94	0.10
Selected maternal comorbidities **								
Any	2.22	0.99	5.36	0.06	2.05	0.84	5.28	0.12
CHD	0.73	0.04	4.09	0.77	0.32	0.02	2.23	0.32
DM	0.87	0.28	2.28	0.80	0.96	0.28	2.82	0.94
Obesity	4.63	2.07	10.64	0.001	3.61	1.44	9.46	0.01

* Adjusted for race/ethnicity, COVID-19 immunization, insurance, comorbidities, and disease severity.

** CHD: congenital heart disease; DM: diabetes mellitus; REF: reference; Asymp: asymptomatic.

**Table 4.** Race associations with adverse outcomes (AP) in patients with asymptomatic/mild/moderate COVID-19.

Variable	OR	Crude 95% CI		*p*-Value	OR	Adjusted * 95% CI		*p*-Value
Ethnicity								
White (Ref)	REF							REF
Asian	0.86	0.31	2.30	0.77	0.86	0.30	2.33	0.77
Black/African American	4.35	1.11	21.78	0.05	4.14	1.02	21.22	0.05
Hispanic/Latina	0.95	0.47	1.98	0.88	0.89	0.41	1.92	0.77
Unknown/other	1.02	0.38	2.69	0.97	1.12	0.40	3.11	0.83

* Adjusted for COVID-19 immunization, insurance, and comorbidities.

## Data Availability

The data presented in this study are available on request from the corresponding author due to privacy considerations.
